# Differential diagnosis of tuberculous meningitis from partially-treated pyogenic meningitis by cell ELISA

**DOI:** 10.1186/1471-2377-4-16

**Published:** 2004-10-22

**Authors:** Rajpal S Kashyap, Rani P Kainthla, Ravindra M Satpute, Neha P Agarwal, Nitin H Chandak, Hemant J Purohit, Girdhar M Taori, Hatim F Daginawala

**Affiliations:** 1 Biochemistry Research Laboratory, Central India Institute of Medical Sciences, 88/2 Bajaj Nagar, Nagpur-440010, India; 2 Environmental Modeling and Genomic division, NEERI, Nehru Marg, Nagpur-440020, India

## Abstract

**Background:**

Tuberculous meningitis (TBM) is a major global health problem, and it is sometimes difficult to perform a differential diagnosis of this disease from other diseases, particularly partially-treated pyogenic meningitis (PTPM). In an earlier study, we demonstrated the presence of a 30-kD protein antigen in cerebrospinal fluid (CSF) of TBM patients. We have also shown that lymphocytes from CSF of TBM patients respond differently to this antigen than do those from PTPM patients. The purpose of this study was to develop an assay that can discriminate between TBM and PTPM.

**Methods:**

We developed a cell enzyme-linked immunosorbant assay (Cell ELISA) to quantitatively measure production of antibodies against the 30-kD protein in B cells from CSF of TBM and PTPM patients.

**Results:**

The cell ELISA yielded 92% (11/12) sensitivity and 92% (11/12) specificity for the differential diagnosis of TBM from PTPM.

**Conclusion:**

When induced with the 30-kD protein antigen, B cells derived from CSF of TBM patients respond to IgG production within 24 h while those derived from PTPM patients do not respond.

## Background

Tuberculous meningitis (TBM) is an infection of the central nervous system (CNS) that is prevalent in both under-developed and developing countries. An increased incidence of TBM has occurred in recent years due to the growing number of people infected with human immunodeficiency virus (HIV). Diagnosis of TBM remains problematic despite many new, advanced diagnostic methods [1,2]. Previous clinical studies have clearly demonstrated that the timing of TBM treatment is the most critical factor in determining the ultimate outcome, which underscores the importance of early diagnosis [3]. The laboratory confirmation for the diagnosis of TBM is based on the detection of acid-fast bacilli (AFB) in the cerebrospinal fluid (CSF) and by culturing CSF for Mycobacterium tuberculosis bacilli (MTB) [4]. However, the sensitivity of direct AFB smears from CSF ranges from 5–10% and culturing techniques take 4–6 weeks. It has been recently reported that the staining efficiency of the AFB smear test can be increased to detect up to 50% of TBM cases, but this technique requires a very large amount of CSF [5].

Clinical as well as CSF features are helpful for diagnosing TBM, but they cannot be used to differentiate TBM from other infectious and non-infectious disorders [6,7]. In particular, clinicians often encounter difficulty when performing a differential diagnosis of TBM from partially-treated pyogenic meningitis (PTPM) cases. Both the results from biochemical and pathological analysis of CSF and the clinical presentation of TBM are often similar to those of PTPM, which results in frequent misdiagnosis.

In an earlier study, we reported the presence of a diagnostic 30-kD protein antigen in CSF of confirmed and suspected TBM patients [8]. Immunological methods such as antibody-capture enzyme-linked immunosorbant assay (ELISA) have been previously used for diagnosing TBM [9]. The cell ELISA method allows further confirmation of the results obtained by antibody-capture ELISA.

Cellular immune function is characterized by the existence of various types of lymphoid cells. As lymphocytes participate in the production of humoral immunity, they may respond to the 30-kD protein antigen in TBM and PTPM patients. We have developed a cell ELISA to study the response of B cells derived from CSF of TBM and PTPM cases following challenge with the 30-kD protein antigen. The purpose of the present study was to evaluate the antibody response to the 30-kD protein antigen in CSF of TBM and PTPM patients by cell ELISA and to determine whether this method may be used in differential diagnosis of TBM from PTPM.

## Methods

### Patients and sample collection

The Central India Institute of Medical Sciences (CIIMS), Nagpur, is a tertiary referral center. CSF was collected from patients who were suspected of having TBM or other infections before they received any treatment. For patients undergoing cranial surgery, analysis of CSF was performed if they were suspected of having meningitis. These patients were already on broad-spectrum antibiotics, such as third-generation cephalosporins and aminoglycosides.

To establish a diagnosis of meningitis, 2–5 ml CSF was withdrawn from patients using a lumbar puncture. CSF was then subjected to routine biochemical analysis and pathological analysis including Gram staining, India ink staining, and AFB staining and culturing. One milliliter of CSF was used for the cell ELISA study, and 1 ml was used for detection of the 30-kD protein band by SDS-PAGE analysis in 12 randomly selected TBM and PTPM patients. Diagnosis of TBM and PTPM was based on the criteria described below.

### Diagnostic criteria

#### 1. Tuberculous Meningitis (TBM)

Presence of Mycobacterium tuberculosis in CSF by staining and/or culture, OR

Clinical meningitis with the following observations:

A. Sub-acute or chronic fever with features of meningeal irritation such as headache, neck stiffness, and vomiting with or without other features of CNS involvement

B. CSF findings showing increased proteins, decreased glucose (CSF:blood glucose ratio <0.5), and/or pleocytosis with lymphocytic predominance

C. Presence of the 30-kD protein band in CSF on SDS-PAGE analysis

D. Good clinical response to antituberculous drugs

None of the 12 TBM patients had positive AFB staining.

#### 2. Partially-treated pyogenic meningitis (PTPM)

Presence of pathogenic bacteria in CSF by staining and/or culture, OR

Clinical meningitis with the following observations:

A. Fever and/or signs of meningeal irritation (patients who have undergone cranial surgery to treat tumor(s), stroke, or head injury and who have received antibiotics), OR High fever and/or signs of meningeal irritation with or without CNS manifestations (patients who received broad-spectrum antibiotics)

B. CSF findings showing increased proteins, decreased glucose (CSF:blood glucose ratio <0.2), and/or pleocytosis with a predominance of polymorphonuclear cells; CSF may resemble that of chronic meningitis patients

C. Absence of the 30-kD protein band in CSF on SDS-PAGE analysis

D. Good clinical response to broad-spectrum antibiotics

#### 3. Control group

Peripheral blood samples from six healthy volunteers were also analyzed and included as negative controls.

### Laboratory studies

#### Sodium dodecyl sulfate polyacrylamide gel electrophoresis (SDS-PAGE

CSF samples obtained from confirmed and suspected TBM cases were subjected to SDS-PAGE. SDS-PAGE was performed with a vertical slab gel electrophoresis system (Broviga, India) using the standard Laemmali method (10). A 4% stacking gel and 10% running gel were used. Electrophoresis was carried out at 250 volts/50 mAmps. Gels were developed by staining with Coomassie brilliant blue GR-250 and the protein profiles were then studied. Band size (i.e., molecular weight) was estimated using molecular weight markers (Genei, Bangalore, India) in a parallel lane.

#### Antigen (30-kD) preparation

Following separation of proteins from CSF of confirmed TBM patients (AFB-positive) by SDS-PAGE, the 30-kD protein band was sliced out of the gel and pre-equilibrated in elution buffer (0.15 M phosphate-buffered saline [PBS], pH 7.4) and then electro-eluted in a whole gel eluter system (Biotech, India) for 90 min at 30 volts (11). The sample was then harvested from the unit and dialyzed against PBS and the protein content was measured using a Bio Lab KIT. Protein purity was checked using native PAGE and was then used to evaluate the antibody response of B cells derived from CSF of TBM and PTPM patients.

#### Preparation of CSF Cells

One milliliter of CSF collected from TBM and PTPM patients was centrifuged at 400 rpm for approximately 20 min. The supernatant was then discarded and the cell pellet was washed two times with PBS and then diluted in RPMI 11640 tissue culture medium containing 10% fetal calf serum.

#### Preparation of Blood Cells

Heparinized blood samples were obtained from six healthy volunteers. Peripheral blood mononuclear cells (PBMC) were isolated from heparinized blood by standard Ficoll-Hypaque gradient centrifugation. PBMCs were dissolved in PBS and centrifuged at 400 rpm for approximately 15–20 min, and the PBMCs were then diluted in RPMI 11640 tissue culture medium containing 10% fetal calf serum.

#### Cell ELISA

Flat-bottomed, 96-well ELISA plates were coated with 10 μg 30-kD antigen/ml diluted in PBS (pH 7.2). Following overnight incubation, the plates were washed with PBS and then coated with 5% BSA-PBS for 4 h. The plates were then washed five times with PBS. Two-hundred μl of the cell preparation derived from CSF of patients with TBM or PTPM were then added to the wells and coated. Each sample was prepared in duplicate. Plates were maintained overnight at 37°C in 5% CO_2 _in a carbon dioxide incubator. The following day, the plates were washed with PBS and horseradish peroxidase (HRP)-conjugated rabbit anti-human IgG (1:10,000) was then added to the plates. After a 2-hr incubation at 37°C, the plates were washed again with PBS and 100 μl tetramethylbenzidine (TMB)/H_2_O_2 _were added. The TMB/H_2_O_2 _served as a substrate for HRP. After a 15-min incubation, 100 μl stop solution (2.5 N sulphuric acid) were added and the plates were then read with an ELISA reader at 450 nm (12).

## Results

**Figure 1:**
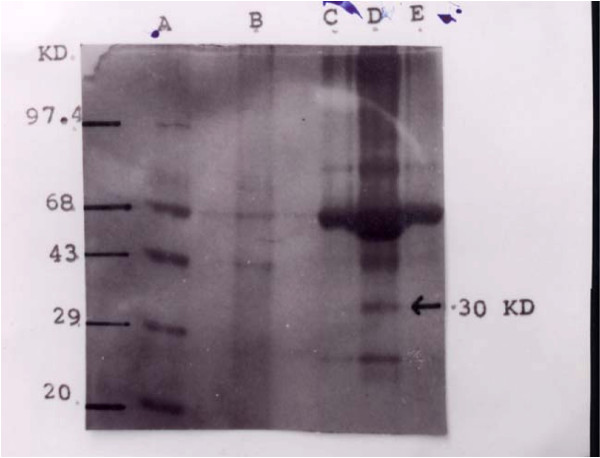
SDS-PAGE electrophoretogram of CSF from control (lanes B, C, E) and suspected (lane D) TBM subjects. Molecular weight marker is shown in lane A. The arrow indicates the 30-kD band, which represents the 30-kD protein antigen

Detailed clinical data for TBM and PTPM patients are presented in Table 1. Out of the 12 PTPM patients, two cases harbored microorganisms, which were cultured (gram-positive cocci in one case and gram-negative bacilli in the other case). Among the 12 patients who fulfilled the criteria for TBM (shown in Table 1), CSF of all these patients was positive for the 30-kD protein antigen and was negative for AFB. None of the patients had a previous history of extra-CNS tuberculosis. In addition to the patients described in Table 1, we also tested an additional 700 CSF samples, including 150 from TBM patients. The 30-kD protein antigen was observed in >90% of these TBM patients (data not shown). Figure 1 shows the presence of the 30-kD protein band in the CSF of suspected TBM cases. This band was markedly absent from the CSF of PTPM patients.

**Table 1:** Clinical and CSF Findings for TBM and PTPM Patients

	**CSF Analysis**									
**Case No.**	**Age (years)/Sex**	**TLC**	**P %**	**L %**	**Protein (mg/dl)**	**Sugar (mg/dl)**	**CSF:blood sugar ratio**	**Neck Stiffness**	**Duration of fever (weeks)**	**Headache**

**TBM**										

1*	16/f	30	-	100	83	27	0.32	Present	16	Present
2	8/m	90	5	95	105	30	0.31	Absent	8	Present
3	54/f	25	2	98	47	67	0.54	Absent	3	Present
4	16/f	450	18	82	535	21	0.24	Absent	12	Present
5*	26/m	120	60	38	143	43	0.66	Present	12	Present
6	58/m	14	-	100	68	37	0.44	Present	-	Absent
7	31/f	180	12	88	203	86	0.53	Absent	1	Present
8	55/f	112	10	90	231	22	0.38	Present	4	Present
9	65/m	150	1	99	131	23	0.33	Present	4	Present
10	56/m	121	12	88	217	41	0.47	Present	2	Present
11	7/f	32	35	65	68	31	0.32	Present	4	Present
12	43/f	60	-	100	97	39	0.48	Present	2	Present
**PTPM**										
1^#^	38/m	220	90	10	96	26	0.20	Present	1	Present
2^#^	63/f	1600	61	38	401	15	0.14	Present	1	Present
3**#	23/m	61	95	5	270	33	0.20	Absent	6	Present
4^+π^	25/m	180	88	12	203	86	0.53	Present	1	Present
5^#^	48/m	450	77	32	868	32	0.21	Present	8	Present
6^π^	14/f	150	84	11	61	38	0.31	Absent	1	Present
7^ψ^	52/m	140	92	8	471	12	0.14	Absent	-	Absent
8^#^	56/m	430	80	20	518	17	0.22	Absent	-	Present
9^++ψ^	6/m	36	82	9	61	107	0.33	Absent	-	Absent
10^#^	62/f	40	73	27	142	25	0.25	Absent	-	Present
11^#^	4/m	50	90	5	71	24	0.19	Absent	-	Absent
12^π^	27/m	200	78	22	131	21	0.18	Absent	2	Present

%P- Polymorphs, % L- Lymphocytes.

*Pulmonary tuberculosis (on chest x-ray) **Consolidation (on chest x-ray)

^#^Cranial surgery post operative. ^ψ ^Post Head injury, ^π ^Presented to CIIMS as PTPM.

^+ ^gram-negative bacilli observed, ^++ ^gram-positive& non-capsulated cocci pairs observed.

**Figure 2:**
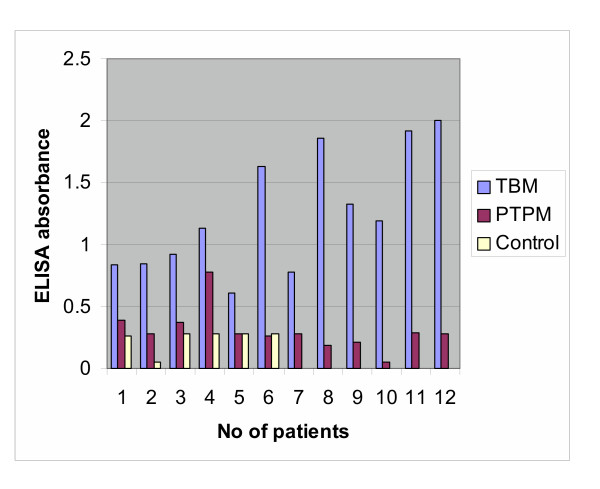
B Cell response (IgG reactivity) to the 30-kD protein antigen in CSF cells derived from tuberculous meningitis (TBM) and partially-treated pyogenic meningitis (PTPM) patients and peripheral blood cells from control subjects

The ELISA absorbance values of IgG to the 30-kD protein antigen in CSF from TBM and PTPM patients are presented in Figure 2. The cut-off value (OD at 450 nm) for positivity to the 30-kD protein antigen IgG in the control CSF is 0.6. High-titer values for IgG antibody production against the 30-kD protein antigen were observed in 11 out of 12 TBM patients. However, the titer in PTPM patients was much lower than that observed in TBM patients. IgG antibody production (expressed as ELISA absorbance value) ranged from 0.7 to 2.0 for cells derived from CSF of TBM patients, with the exception of case no. 5 (ELISA absorbance value, 0.59), and from 0.05 to 0.38 for cells derived from CSF of pyogenic meningitis cases, with the exception of case no. 4 (ELISA absorbance value, 0.79). The sensitivity of the cell ELISA was 92% and the specificity was 92% for differential diagnosis of TBM from PTPM. No IgG antibodies to the 30-kD protein antigen were produced by PBMCs from six healthy individuals within 48 h of exposure to the 30-kD protein antigen.

## Discussion

During the past decade, several conventional immunoassays including ELISA, dot immunobinding assays, immunoblot assays, and various molecular methods such as the polymerase chain reaction (PCR) have been reported as adjuncts in the diagnosis of TBM [4,13-15]. However, difficulties have been encountered when using many of the aforementioned techniques to differentiate TBM from PTPM. CSF TLC, DLC (total and differential leukocyte count), protein, and glucose estimation are helpful parameters for establishing a TBM diagnosis and for differentiating other infectious and non-infectious neurological disorders, but these tests are non-specific and often cannot differentiate TBM from PTPM in patients in whom organisms are not observed. Delays in diagnosis and treatment are regarded as major contributing factors to the high mortality and morbidity of TBM, and any delay in starting appropriate medication for TBM and PTPM worsens the outcome.

We previously used SDS-PAGE to demonstrate the presence of a 30-kD protein antigen in the CSF of TBM patients that is specific to *M. tuberculosis *and may be considered to be a diagnostic marker for TBM. In this study, we used this 30-kD protein antigen to evaluate the IgG antibody response of B cells derived from CSF of TBM and PTPM patients and from peripheral blood samples from six healthy volunteers. A cell ELISA was developed for the quantitative measurement of antibody production against the 30-kD protein antigen by these cells. Higher titers of IgG antibody production were observed in TBM patients compared to PTPM patients. The cells obtained from CSF of TBM patients gave an early response, presumably because they were already sensitized against the TBM antigen. However, when challenged with the 30-kD protein antigen, the cells obtained from PTPM patients and healthy volunteers gave a delayed response since they are not sensitized against this antigen. Therefore, an early response on this time scale is indicative of TBM.

We have thus shown that cell ELISA is a sensitive technique for the differential diagnosis of TBM from PTPM. This method involves the demonstration of active antibody production by cells, particularly those derived from the affected site [16]. Previously, we standardized cell ELISA methodology in our laboratory using standard culture filtrate protein of *M. tuberculosis *(H37Rv strain) received from Colorado State University, Fort Collins USA (data not shown). The only limitation of this method is the time period (24–30 h) involved. However, the sensitivity of the test overcomes this drawback since it the only reported method that can discriminate TBM from PTPM.

The sensitivity and specificity of IgG antibody in differential diagnosis of TBM from PTPM using the 30-kD protein antigen by cell ELISA was found to be 92% (11/12). We have also demonstrated that antibody production against the 30-kD protein antigen is higher in cells derived from CSF of TBM patients compared to PTPM patients.

Various methods have been developed in our laboratory that yield a high specificity and sensitivity for diagnosis of TBM, but a small number of false positive results have been observed in pyogenic meningitis cases, particularly PTPM cases [17,18]. The cell ELISA method developed in our laboratory using the 30-kD protein antigen marker can potentially provide additional information to the treating physician that may enable a differential diagnosis of TBM from PTPM.

The cell ELISA method for diagnosing TBM is based on the assumption that local synthesis of humoral antibodies against MTB antigen occurs. Various researchers have shown that CSF-derived cells have a significantly higher proliferation response to purified protein derivative (PPD) in patients with TBM, which is suggestive of an intrathecal immune response [11,19].

Our data can be summarized by the following observations: first, cell ELISA is a useful method for differentiating TBM from PTPM using the 30-kD protein antigen; second, the method of challenging B cells from CSF of suspected TBM patients with the 30-kD protein antigen can be helpful in confirming a TBM diagnosis; and third, the cell ELISA allows several samples to be analyzed simultaneously. Hence, the cell ELISA should be a very useful tool for the differential diagnosis of TBM from PTPM.

## Conclusion

The presence of a 30-kD protein antigen in CSF of TBM patients indicates that this protein carries the candidate marker antigen which is specific to *M. tuberculosis*. We have demonstrated that by using cell ELISA, we can differentiate TBM patients from PTPM patients, which should be helpful for diagnosing TBM. Additionally our results suggest that lymphocytes from CSF of TBM patients when challenged with 30 kD protein give a quick response by producing IgG antibodies when compared with that of PTPM and healthy volunteers. This may be because lymphocytes from TBM patients have already been exposed to 30 kD MTB antigens.

## Competing interests

The authors declare that they have no competing interests.

## Authors' contributions

RSK carried out the study design, data collection, statistical analysis, data interpretation, literature search, and manuscript preparation; NPA, RPK, and RMS assisted in data analysis collection; NHC assisted in data collection, statistical analysis, and data interpretation; HJP participated in the preparation of the manuscript, data interpretation, and study design; GMT provided assistance in preparation of the manuscript, data interpretation, study design, and funds collection; and HFD supervised the study design, statistical analysis, data interpretation, manuscript preparation, and literature search.

## Pre-publication history

The pre-publication history for this paper can be accessed here:


